# The Overview Effect and Creative Performance in Extreme Human Environments

**DOI:** 10.3389/fpsyg.2021.584573

**Published:** 2021-03-12

**Authors:** William Frank White

**Affiliations:** ^1^Division of Continuing Education, Harvard University, Cambridge, MA, United States; ^2^Human Factors for Space Settlement, Kepler Space Institute, Tallahassee, FL, United States

**Keywords:** overview effect, creativity, earth gazing, cupola, astronauts

## Introduction

We tend to think of creativity as a trait that takes someone outside the norms of everyday behavior.

A typical definition of creativity would be:

The use of the imagination or original ideas, especially in the production of an artistic work^1^.

Creative performance stands in contrast to, say, routine performance. As a simple example, consider working on an assembly line. The objective is not to dream up a new way to place seats in a car, but rather to place all the seats in the car, in the same way, every time. Saying this does not denigrate the dignity and value of the work; it simply states a fact about the degree of creativity it requires. Moreover, the more routine the job, the more likely it is to be automated.

By comparison, consider a classic example of creativity: painting. If I, as an artist, paint the same scene over and over again, as if I am on an assembly line, very few people would consider me to be “creative.” Even more to the point, if I paint a scene that is imitative of another artist's work over and over again, I would not be considered creative. I would be seen as unoriginal at best and a forger at worst.

Our society depends on citizens who are willing to perform routine tasks and those who seek original means of expressing themselves. We could not survive if our genes and our social systems tilted too far in one direction or the other.

This paper considers creative performance in extreme environments, with a focus on astronauts. We begin with creative performance and the environment, then turn to explorers in general, and conclude with astronauts specifically.

## Creative Performance and the Environment

Does the environment make a difference in fostering or inhibiting creative performance? This question is worth asking when we consider its relevance on Earth, but it becomes especially pertinent when examining astronaut performance. Everything an astronaut does takes place in an environment that is radically different from the terrestrial surroundings in which humanity evolved.

Dul makes the point that, in fact, relatively little research has been conducted on the physical environment's impact on creativity, with most studies focusing on the social environment. In a survey of 44 studies, only one considered the physical environment (Dul, 2019). Dul offers a “triple-path theoretical framework” suggesting that the objective physical environment is related to creativity through three perceptual paths: functionality, meaning, and mood (Dul, 2019). This structure offers a helpful beginning that may eventually be relevant to the space environment.

Amabile is well-known for her focus on the social environment and the difference between extrinsic rewards and intrinsic satisfaction in supporting creativity. In the case of astronauts, we see the importance of the social environment as it influences creative performance (Amabile, 2018).

Professional astronauts are unique in this regard because, for the most part, they have been striving for years to achieve the goal of simply becoming an astronaut. In that sense, they do not require any external rewards to be satisfied by what they are doing. At the same time, they are part of a team that has a mission, and they are supported by an even larger group that oversees and evaluates their performance. Therefore, they may represent a population motivated by both internal and external factors.

Ultimately, it must be noted that the space environment is fundamentally different from almost any terrestrial environment. No matter where one goes on Earth, normal gravity is present, as is air that can (usually) be breathed without danger, and harmful radiation is mediated by the Earth's atmosphere and magnetosphere.

In space, astronauts live in a weightless world, the air they breathe is artificially produced by their spaceship or space station, and radiation is a constant concern. They are literally risking their lives throughout their missions.

There are, of course, extreme environments on Earth, both natural and artificial. The summit of Mount Everest is a place where very few climbers can go without taking oxygen with them. Scott Parazynski, the only person who has both been in outer space and summited Everest, told me that he was more concerned for his safety on the mountain than while in orbit^2^. He said that was because large teams of people support astronauts when they are on the International Space Station, and its interior is a shirt-sleeve environment. When a climber is on Everest, however, the support teams are much smaller and the mountain seems much closer and more threatening.

## The Societal Role of Explorers

Examining the historical record suggests that human beings have always been explorers, or at least, that a significant portion of the species has been. This means that they seek out environments where creativity is required of them. While family and friends may remain home, where daily events are relatively predictable, explorers, by definition, do not know what they might face as they move deeper into uncharted territory.

Returning to our definition, this requires “using imagination or original ideas…” If you are Sir Ernest Shackleton and your Antarctic expedition's ship becomes frozen, then crushed, in pack ice that stretches as far as the eye can see, your original plan for the journey has to be thrown out and you have to think in new ways about what to do next. In other words, you need to resort to your imagination for original ideas to survive. The story of Shackleton's response to this potential disaster is too complex to review here, but suffice it to say that he managed to rescue every one of the members of his expedition through his creative and courageous actions.

Fast-forward 100 years from the days of polar expeditions and we find the paragons of modern exploration, the astronauts. They share many common characteristics with their predecessors, but are unique in the annals of exploration because there is no more extreme environment for human beings, indeed for life itself, than outer space. Whether in the deserts of North Africa, the jungles of Southeast Asia, or the glaciers of Antarctica, explorers have air, water, and gravity that support their quests. But human beings cannot survive once they reach a certain altitude above the Earth's surface, where “space” begins. Only through artificial means can astronauts carry out their missions. What does this mean for creative performance? What contributes to creativity in space and what blocks it?

## Creative Performance Through Preparation

In his book, *An Astronaut's Guide to Life on Earth*, Canadian astronaut Chris Hadfield suggests that being an astronaut is a way of life that is not limited to the time spent in outer space. He points out that, because of the extreme environment of space, every moment an astronaut spends there puts his or her life in danger. For that reason, much of an astronaut's time is actually focused on preparation for flight and simulating every possible situation the crew might encounter. While on the International Space Station (ISS), the astronaut is away from family and friends, isolated from the vast majority of human beings and normal life (Hadfield, 2013).

Space travelers cannot simply walk outside for a breath of fresh air when they feel isolated or stressed. They are separated from nature and the healing effects of walking in the woods or hiking in the mountains. Their immediate social contacts are limited to the other crew members and they work very hard during their missions.

What impact might all of these elements have on creative performance?

Let's look first at how astronauts prepare for space missions.

Weighing multiple options in response to unpredictable situations should free the mind up to be more creative. In fact, that is why astronauts simulate so many alternatives: they never know exactly when they might have to improvise with very little time available. And when they do, there is even less time to stop and think about what to do. Seconds count, and the response becomes instant and instinctive, because the stimulus has been seen before in the simulations.

At another level, the underlying awareness of constant danger, coupled with the boredom that accompanies isolation might actually support artistic creativity (Peldszus et al., 2014)^3^. As one example, consider Chris Hadfield's cover of David Bowie's classic, “A Space Oddity.” Playing the guitar while floating weightless throughout the International Space Station, Hadfield does a credible job of creating a watchable music video. Nicole Stott had begun painting before her astronaut career, but has been working almost full-time as an artist since her retirement from NASA^4^.

## Creative Performance and the Overview Effect

There is another unique aspect of space exploration (i.e., seeing the Earth and the universe from a vantage point that no human experienced before 1961, when Yuri Gagarin went into orbit). The “Overview Effect” (a term coined by the author) has positive psychological benefits for astronauts, balancing some of the inherent challenges of spaceflight. Astronauts on the ISS enjoy going to the “cupola” and practicing “Earth gazing” during their free time. They say it brings a shift from identifying with parts of the Earth to identifying with all of it. They also emphasize that the Earth is always doing something new, so that the experience never becomes dull or boring. Increasingly, astronauts are describing the Earth as a “living being” (White, 2014).

Experiencing the Overview Effect also seems to bring a sense of calm and tranquility to the viewer, which may offset some of the more stressful aspects of spaceflight. This is a topic that has not been covered in great detail in the literature about spaceflight or the Overview Effect, but it is worthy of consideration. We hear quite a lot about the astronaut workload when they are on missions, including time on the ISS, which is somewhat less pressured than Shuttle flights. However, we know that “downtime” is important for Earthlings and that creative moments often arise when people are relaxing in various ways (National Aeronautics Space Administration, 2019).

While negative incidents in space have rarely been documented, there have been cases of Mission Control attempting to keep astronauts to a tight script and of astronauts rebelling against the limits placed on them. It costs a huge amount of money to put Earthlings into orbit and space agencies naturally want to generate a robust ROI, so the Mission Control intentions are understandable. On a positive note, this attitude has changed with the advent of long-duration ISS missions.

## Two Sides of the Same Coin

Constantly simulating potential problems and relaxing while floating in zero-G and gazing out at the Earth may seem like polar opposites, but perhaps they are two sides of the same coin.

One of the newest additions to the International Space Station is the cupola, a special viewing area that allows for a 360-degree view of the Earth and the universe while floating gently above the cupola window. We know from their own reports that astronauts spend as much time in this setting as possible and seem drawn to it during their “downtime.”

Annahita Nezami, Ph.D., a psychologist who wrote her PhD thesis on the Overview Effect, is a collaborator of mine on several projects. Based on her findings and past research, Nezami states:

Earthgazing can motivate positive psychological growth by the way it diminishes cognitive and emotional stress and cultivates positive emotions such as belonging, connection, gratitude, reverence, awe, and humility (Meier et al., 2020).

Nezami suggests that “Earth gazing has the potential to create inner harmony and help us reconsider societal values, while promoting psychological and societal well-being^5^.”

The cupola's history on the International Space Station is worth exploring in terms of its intentions. Some descriptions suggest that it was added for reasons other than Earth gazing, but it seems that astronaut relaxation has become a primary function.

When astronauts talk about their experiences of looking at the Earth, their language is often meditative and contemplative. It seems that Earthgazing fulfills many of the functions of meditation and relaxation on the surface of the planet.

For example, payload specialist Byron Lichtenberg described how he would float up to the cockpit after his shift on the Space Shuttle was over and “watch the world go by” while eating dinner:

Thinking about eating your dinner around the world in 90 min is really something. I took advantage of that time; then I would stay up and look for another half-orbit and end up going to bed exhausted (see text footnote 5).

During the pre-ISS spaceflight days, each mission was packed with long “to do lists,” and Shuttle astronauts like Lichtenberg had to carve out the time to do any significant Earthgazing (which may be why he went to bed “exhausted”). However, much has changed with the advent of the ISS. Astronauts still work long hours during the week, but mission planners have begun to recognize that these extraordinary people are still human and they need time off to rest, relax, communicate with their families, and, yes, experience the Overview Effect^6^.

In the words of Nicole Stott, the experience is much different with the extra time and different view provided by the cupola:

It is a really impressive place and it has changed the view that you get from flat, Earth-facing windows. Honestly, it was so much more impressive than we expected. You are always looking for the horizon, and you could find it before, but the cupola really puts it right in your face. You really get the curvature of the Earth, and you get much more of a feeling of a planet hanging in space (Canadian Space Agency, 2020).

For years, we have considered the primary job of astronauts to be completing as many scientific experiments as possible during their time in orbit. It has taken a long time for space agencies and the public to see the Overview Effect as a benefit not only to the astronauts but also to surface dwellers because it provides a new perspective on our planet and our species.

However, the value of the Overview Effect as a shift in worldview has only recently been seen as actually supporting that other goal of completing work in orbit. This discovery offers compelling lessons for the future of space training and space architecture: we should really think about astronaut leisure time and build in opportunities for relaxation and Earthgazing^7^.

The result will likely be more productivity and greater creativity, when it counts. Since the astronauts are living and working on the ISS as our representatives, we can expect that this shift in approach will benefit the people of Earth as well.

Are we able, at this time, to provide empirical evidence that Earth gazing actually improves creative performance by the astronauts? No, but perhaps this examination of the potential relationship between the Overview Effect and performance will encourage additional study to determine if there is, in fact, a connection.

## Author Contributions

The author confirms being the sole contributor of this work and has approved it for publication.

## Conflict of Interest

The author declares that the research was conducted in the absence of any commercial or financial relationships that could be construed as a potential conflict of interest.

## Footnotes

^1^*Google Dictionary*.

^2^Interview with Frank White for fourth edition of *The Overview Effect*, to be published in 2021 by Multiverse Publishing.

^3^Hadfield: Available online at: https://www.youtube.com/watch?v=KaOC9danxNo

^4^Nezami, Unpublished proposal for a book with Frank White, 2019.

^5^*The Overview Effect*, 210.

^6^*The Overview Effect*, 305.

^7^This would be a valuable area of research for NASA to undertake, especially when considering space exploration of the Moon and Mars.
